# A Novel Fibrosis Index Comprising a Non-Cholesterol Sterol Accurately Predicts HCV-Related Liver Cirrhosis

**DOI:** 10.1371/journal.pone.0093601

**Published:** 2014-04-03

**Authors:** Magdalena Ydreborg, Vera Lisovskaja, Martin Lagging, Peer Brehm Christensen, Nina Langeland, Mads Rauning Buhl, Court Pedersen, Kristine Mørch, Rune Wejstål, Gunnar Norkrans, Magnus Lindh, Martti Färkkilä, Johan Westin

**Affiliations:** 1 Department of Infectious Diseases/Virology, Institute of Biomedicine, University of Gothenburg, Gothenburg, Sweden; 2 Department of Mathematical Sciences, Chalmers University of Technology and University of Gothenburg, Gothenburg, Sweden; 3 Department of Infectious Diseases, Odense University Hospital, Odense, Denmark; 4 Department of Clinical Science, University of Bergen, Bergen, Norway; 5 Department of Infectious Diseases, Aarhus University, Aarhus, Denmark; 6 Institute of Clinical Medicine, Department of Gastroenterology, Helsinki University, Helsinki, Finland; Rosalind Franklin University of Medicine and Science, United States of America

## Abstract

Diagnosis of liver cirrhosis is essential in the management of chronic hepatitis C virus (HCV) infection. Liver biopsy is invasive and thus entails a risk of complications as well as a potential risk of sampling error. Therefore, non-invasive diagnostic tools are preferential. The aim of the present study was to create a model for accurate prediction of liver cirrhosis based on patient characteristics and biomarkers of liver fibrosis, including a panel of non-cholesterol sterols reflecting cholesterol synthesis and absorption and secretion. We evaluated variables with potential predictive significance for liver fibrosis in 278 patients originally included in a multicenter phase III treatment trial for chronic HCV infection. A stepwise multivariate logistic model selection was performed with liver cirrhosis, defined as Ishak fibrosis stage 5–6, as the outcome variable. A new index, referred to as Nordic Liver Index (NoLI) in the paper, was based on the model: Log-odds (predicting cirrhosis) = −12.17+ (age×0.11) + (BMI (kg/m^2^)×0.23) + (D_7_-lathosterol (μg/100 mg cholesterol)×(−0.013)) + (Platelet count (x10^9^/L)×(−0.018)) + (Prothrombin-INR×3.69). The area under the ROC curve (AUROC) for prediction of cirrhosis was 0.91 (95% CI 0.86–0.96). The index was validated in a separate cohort of 83 patients and the AUROC for this cohort was similar (0.90; 95% CI: 0.82–0.98). In conclusion, the new index may complement other methods in diagnosing cirrhosis in patients with chronic HCV infection.

## Introduction

Morbidity and mortality in Hepatitis C virus (HCV) infection results mainly from the development of complications of cirrhosis, or hepatocellular carcinoma (HCC) [1]. Thus, the assessment of liver fibrosis is of pivotal importance. Although the development of new, highly efficacious treatment regimens will likely lessen the significance of fibrosis staging for prognostication of treatment outcome, it may remain central for tailoring of choice and duration of therapy. Additionally, the diagnosis of cirrhosis will continue to be vital to establish the need for HCC surveillance.

Liver biopsy remains the gold standard for the assessment of liver fibrosis [2]. However, due to the potential risk of complications [3] as well as other limitations, e.g. the risk of sampling error and inter-observer variability [4], [5] associated with liver biopsy, several non-invasive methods for fibrosis assessment previously have been proposed. Studies evaluating potential serum biomarkers of fibrosis have been reported, either alone or combined in indices [6]–[16]. Transient elastography has been thoroughly evaluated among HCV infected patients [17]–[20] with a high diagnostic accuracy for cirrhosis [21] but it may often be difficult to obtain a valid examination, especially in obese patients. In recent years algorithms combining different non-invasive methods has improved the diagnostic accuracy for staging of fibrosis [22], [23]. For identification of cirrhosis, transient elastography appears to be the most accurate method when compared with currently available biomarkers [14], [21], but a freely available biochemical index that could complement to liver elastography measurement for the diagnosis of cirrhosis is desirable.

Cholesterol synthesis is a process involving several steps. Lathosterol and desmosterol are intermediates in the cholesterol synthesis pathway and their serum concentration reflects cholesterol synthesis ([24], [25]. Sitosterol, avenasterol and campesterol are plant sterols (phytosterols) derived from ingested food reflecting intestinal absorption, while cholestanol is produced by enzymatic cleavage of endogenous cholesterol. Cholestanol as well as plant sterols reflects biliary secretion, and cholestanol is a marker of chronic cholestasis [26], [27]. In line with this, plasma levels of non-cholesterol sterols have been associated with either chronic cholestasis or hepatocyte function, particularly in the setting of primary biliary cirrhosis (PBC) [28]. Thus, it may be hypothesized that concentrations of such non-cholesterol sterols potentially could serve as biomarkers of disease progression, also in the presence of HCV infection.

The aim of the present study thus was to evaluate a broad range of non-invasive biomarkers for liver fibrosis, including a panel of non-cholesterol sterols (cholestanol, desmosterol, lathosterol, sitosterol and campesterol) in order to create a predictive model for HCV-related cirrhosis. This was done within the framework of a phase III treatment trial, with a well-defined cohort of treatment-naïve patients and where pre-treatment liver biopsies were mandatory.

## Materials and Methods

### Exploratory Set

The exploratory set cohort was derived from a phase III multicenter, investigator initiated, treatment trial for treatment-naïve HCV genotype 2 or 3 infected patients (the NORDynamIC study, n = 382) conducted at 31 centers in Denmark, Finland, Norway and Sweden. Details regarding trial design and outcome have been published previously [29]. The main outcome of the trial was sustained viral response. Evaluation of biomarkers for non-invasive diagnosis of cirrhosis was included among the secondary aims of the trial in the study protocol. All patients included were adults with detectable plasma HCV-RNA of genotype 2 or 3 had compensated liver disease and were seronegative for hepatitis B surface antigen and for antibodies to human immunodeficiency virus (HIV). A liver biopsy within 24 months of entry was required. Body Mass Index (BMI kg/m^2^), alcohol consumption during the previous year (g/week) and duration of infection were recorded. Of the 382 patients enrolled, 298 patients had a liver biopsy that fulfilled the criteria for staging and grading and had serum samples analyzed for potential fibrosis markers in accordance with the study protocol. When all patients with missing data in any of the parameters were excluded 278 patients remained. These patients constituted the exploratory set.

### Validation Set

The validation set was derived from a cohort of 100 chronic HCV infected patients enrolled in a study evaluating the use of liver biopsy, serum fibrosis markers and transient elastography by use of the Fibroscan device [30], performed at the Department of Infectious Diseases at Sahlgrenska University Hospital in Gothenburg in 2008–2010. All consecutive patients referred for a liver biopsy during an eighteen months period were asked to participate. Blood tests for analysis of serum fibrosis markers, transient elastography examination and liver biopsy were performed in the fasting state at the same day. All patients included were HCV-RNA positive, HBsAg negative and anti-HIV negative adults and had signed an informed consent.

Patients with missing data for any of the relevant parameters were excluded from the present study, leaving 83 patients in the validation set.

### Ethical Considerations

#### Exploratory set

Written informed consent was obtained from each participating patient. Ethics committees in each participating country approved the study (i.e. Regional Ethical Review Board, Gothenburg, Sweden (Regionala etikprövningsnämnden i Göteborg), Regional Committee for Ethics in Medical Research, Bergen, Norway (Regionaletisk komite for medisinsk og helsefaglig forskning i Bergen), The Scientific Ethical Committee for the Region of Middle Jutland, Viborg, Denmark (Den Videnskabsetiske Komité for Region Midtjylland), The Scientific Ethical Committee for the Region of South Denmark, Vejle, Denmark (Den Videnskabsetiske Komité for Region Syddanmark), and the Ethics Committee, Department of Medicine for the Hospital District of Helsinki and Uusimaa, Finland (Etiska kommittén för invärtesmedicin)). The study has been registered at the NIH trial registry (ClinicalTrials.gov Identifier: NCT00143000).

#### Validation set

Written informed consent was obtained from each participating patient. The Regional Ethical Review Board, Gothenburg, Sweden (Regionala etikprövningsnämnden i Göteborg) approved the study.

### Serum Markers

In the exploratory cohort, baseline serum samples were drawn within 30 days prior to study entrance. Platelet count (x 10^9/^L) and Prothrombin complex-INR were analyzed at each center. All other serum samples were stored in −70°C and subsequently analyzed at a central laboratory at Helsinki University Hospital, Finland. Liver function tests and serum fibrosis markers analyzed by standard laboratory methods included normalized AST and ALT, Gammaglutamyl-transferase (GGT) (U/L), Bilirubin (μmol/L), Haptoglobin (g/L), alfa2-macroglobulin (g/L), Hyaluronic acid (HA) (ng/ml), amino-terminal propeptide of type III procollagen (PIIINP) (ug/L), Apolipoprotein A1 (g/L) and carboxy-terminal telopeptide of type I collagen (ICTP) and serum cholesterol (mg/100 ml). The non-cholesterol sterols (cholestanol, D8-lathosterol, desmosterol, D7-lathosterol, campesterol, sitosterol, sitostanol, avenasterol and squalene) were analyzed by gas–liquid chromatography (GLC) (Agilent 6890N Network GC System, Agilent Technologies Inc., Wilmington, DE) on a 50-m long Ultra 2 capillary column (5% Phenyl-methyl siloxane) (Agilent Technologies, Wilmington, DE, USA), with 5a-cholestane as internal standard [31], [32].

To correct for differences in serum levels of sterols as a consequence of varying concentrations of lipoprotein particles, the non-cholesterol sterol values are expressed as proportions of serum cholesterol (μg/100 mg of cholesterol) as well as absolute concentrations (μg/100 ml).

In the validation set all serum samples were drawn the same day as the liver biopsy and immediately stored in −70°C. Serum sterols were subsequently analyzed as stated above, all other analyzes were performed according to routine laboratory procedures at the Sahlgrenska University Hospital.

### Histological Assessment

All liver biopsies in the exploratory and validation cohort were retrieved and re-assessed for necroinflammatory activity and fibrosis stage according to the Ishak protocol [33] by two experienced observers in a dual observer consensus fashion as previously described [29] where the two observers first independently scored all biopsies. Potential topics of disagreement were discussed, and a consensus score was agreed upon, which then was used in the analysis. Steatosis was graded as absent (score of 0), mild (score of 1, <30% of the hepatocytes involved), moderate (score of 2, 30–70%) or severe (score of 3, >70%) [34]. Biopsies with a total length of <15 mm or containing less than six portal tracts were excluded. Cirrhosis was defined as Ishak fibrosis stage 5–6.

### Statistical Analysis

The following variables were analyzed in the exploratory set: age, sex, weight, BMI, genotype, platelets, PK-INR, normalized AST and ALT, GGT, Bilirubin, Haptoglobin, alfa2-macroglobulin, HA, PIIINP, Apolipoprotein A1, ICTP, total cholesterol, cholestanol, D8-lathosterol, desmosterol, D7-lathosterol, campesterol, sitosterol, sitostanol, avenasterol and squalene. Classification was made in two groups that encompassed fibrosis stages 0–4 (n = 242) and 5–6 (n = 36), respectively. The suggested classification procedure consisted of two steps: (i) an index that resulted from fitting of a model to the data and (ii) cut-off values for this index that can be used to divide the predictions into two groups.

The data was modeled through logistic regression, with model selection based on Akaike information criterion (AIC). The model selection was done using goodness-of-fit criteria rather than the magnitude of the p-values for each variable. The large number of sometimes highly correlated factors leads to over fitting, i.e. a model that was too large and sensitive to small perturbations in the data. We decided to make the process of model selection more robust by constructing logistic regression for a large number of re-samples of the data and taking only those factors in the final index that appeared in many of these, re-sampled models. The final estimation of the coefficients was performed using the original dataset. The new index based on the model above can be formulated either in terms of probabilities (I_prob_) or log-odds (I_odds_), which are equivalent. I_odds_ uses a more natural scale while I_prob_ may be perceived as more intuitive since it reflects the probability that a certain patient has cirrhosis. I_prob_ was chosen for further calculations, hereafter referred to as the Nordic Liver Index, NoLI. Cutoff values were chosen in order to minimize the misclassification error. Rather than choosing one cut-off, we suggest applying two, C_low_ and C_high_. If the index is below C_low_, the observation will be classified into group 1. If it is above C_high_ it will be classified as group 2. If between C_low_ and C_high_ it will not be classified as either group and further investigations may have to be undertaken to make a correct diagnosis.

Diagnostic performance was analyzed by constructing receiver operator characteristics curves (ROC) for specificity and sensitivity with calculation of the area under the ROC curve (AUROC) and the corresponding confidence interval (CI). The R software package (Free software; www.r-project.org) version 2.15.0 was used for all calculations and the package pROC was used to calculate ROC curves and the corresponding CI. Comparisons of baseline characteristics between groups were analyzed by Mann-Whitney’s U test or Chi-squared test where appropriate. Correlation between transient elastography and NoLI was assessed by Spearman’s r test. These latter analyses were performed using IBM SPSS Statistics version 19.0 software package (IBM Corporation, Somers, NY). All reported p-values are two-sided, and p-values <0.05 were considered significant.

## Results

### Baseline Characteristics

Of the 278 patients included in the exploratory set, 36 (13%) had cirrhosis; baseline characteristics for cirrhotic and non-cirrhotic patients in the exploratory set are reported in Table 1A. Of the 83 patients included in the validation set, 8 (10%) had cirrhosis. Baseline characteristics are displayed in Table 1B.

**Table 1:** Baseline characteristics in the (A) exploratory set and (B) the validation set according to presence of cirrhosis in liver biopsy.

A. Exploratory set	Cirrhosis	Non cirrhosis	P value
	n = 36	n = 242	
Sex female/male (%)	28/72	42/58	n.s
Age (years)	51(46–56)	41 (32–49)	<0.0001
BMI (kg/m^2)^	27 (24–32)	25 (22–28)	0.001
Duration of infection (years)	28.5 (20–35)^a^	11 (6–23)^b^	<0.0001
AST (/ULN)	2.05 (1.53–3.34)	1.23 (0.89–1.94)	<0.0001
ALT (/ULN)	2.32 (1.45–2.83)	1.43 (0.82–2.37)	0.001
Platelet count (10^9^/L)	150 (97–206)	227 (196–266)	<0.0001
Prothrombin index INR	1.1 (1.0–1.2)	1.0 (1.0–1.1)	<0.0001
D7-lathosterol (μg/100 mg cholesterol)	97 (57–127)	118 (87–150)	0.008
Genotype 2/3	33/67	30/70	n.s
Ishak fibrosis stage 0/1/2/3/4 (%)		4/17/35/28/16	NA
Ishak fibrosis stage 5/6 (%)	42/58		NA
**B. Validation set**	**n = 8**	**n = 75**	
Sex female/male (%)	25/75	48/52	n.s
Age (years)	52(44–64)	51(44–55)	n.s
BMI (kg/m2)	27 (25–29)	25(23–28)	n.s
Duration of infection (years)	30(26–40)^c^	30(24–34)^d^	n.s
AST (/ULN)	2.0(1.8– 3.0)	1.1 (0.9–1.8)	0.003
ALT (/ULN)	2.1(1.7–3.6)	1.1 (0.9–1.6)	0.001
Platelets (10^9/^L)	141(109–213)	256(201–299)	0.001
Prothrombin-INR	1.1(1.0–1.2)	1.0(1.0–1.1)	0.02
D7-lathosterol (μg/100 mg cholesterol)	77(47–117)	117(85–155)	0.02
HCV genotype 1/2/3/4/mixed (%)	75/0/12.5/0/12.5	73/1/24/1/1	n.s
Ishak fibrosis stage 0/1/2/3/4 (%)		5/21/40/19/15	NA
Ishak fibrosis stage 5/6 (%)	38/62		NA

All values are presented as median (interquartile range; IQR) or percentage.

a n = 28.

b n = 166.

c n = 6.

d n = 60.

There were no patients with clinically decompensated liver cirrhosis in either exploratory or validation set. Patients in the validation set were older than patients in the exploratory set; median age was 49 (IQR 44–55) vs. 42 years (IQR 34–50) (p<0.001), and had a longer duration of infection; 29 (IQR 24–34) vs. 13.5 years (IQR 6,8–25) (p<0.001) in the validation and exploratory set respectively. In the exploratory set, 69% of the patients were infected with HCV genotype 3, whereas in the validation set HCV genotype 1 was most frequent, present in 72% of patients.

### Model for Prediction of Fibrosis

**Figure 1:**
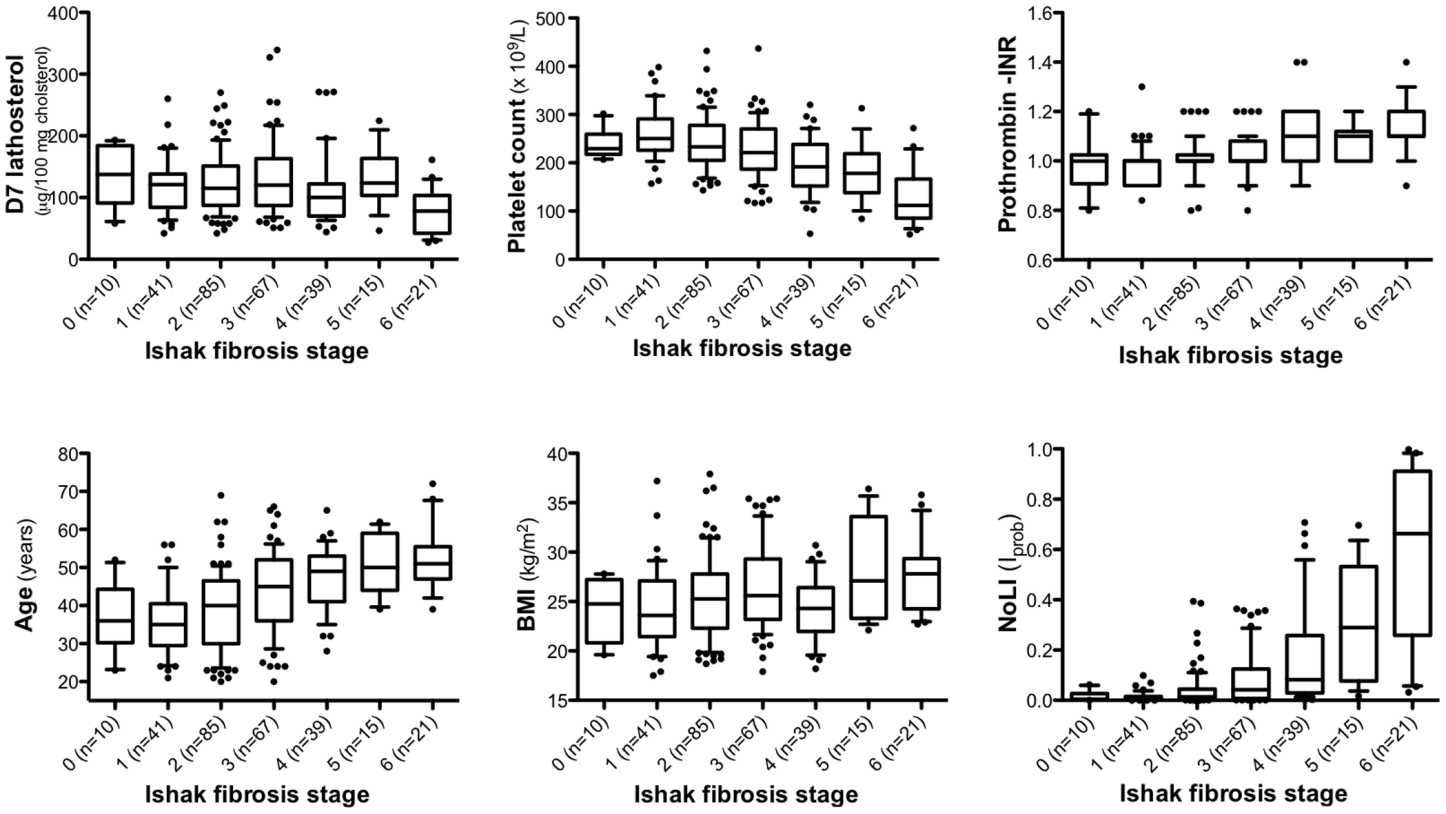
Box-plots displaying the different components of the NoLI index (D_7_ lathosterol, platelet count, Prothrombin complex-INR, age and BMI) and the NoLI index in relation to Ishak fibrosis stage.

Application of the model selection methodology described earlier lead to a final model comprising the following variables: Age (p = 0.0002), BMI (p = 8.0×10^−5^), D_7_-lathosterol (p = 0.02), platelet count (p = 7.0×10^−5^) and Prothrombin-INR (p = 0.122). The model characteristics are summarized in table S1 and the relation between each component of the index separately and Ishak fibrosis stage is displayed in Figure 1. D7 lathosterol, was the only sterol in the panel of sterols, which was independently associated with cirrhosis.

The regression formula for this final model is: Log-odds; I_odds_ (predicting cirrhosis) = −12.17+ (age×0.11) + (BMI (kg/m^2^)×0.23) + (D_7_-lathosterol (μg/100 mg cholesterol)×(−0.013)) + (Platelet count (x10^9^/L)×(−0.018)) + (Prothrombin-INR×3.69).

Predicted probability; I_prob_ = exp (log-odds)/(1+exp (log-odds)).

As stated above, we chose predicted probability (I_prob)_ for further calculations, referred to as the Nordic Liver Index (NoLI). The relation between NoLI and Ishak fibrosis stage is illustrated in the lower right panel of Figure 1.

### Area Under ROC (AUROC)

**Figure 2:**
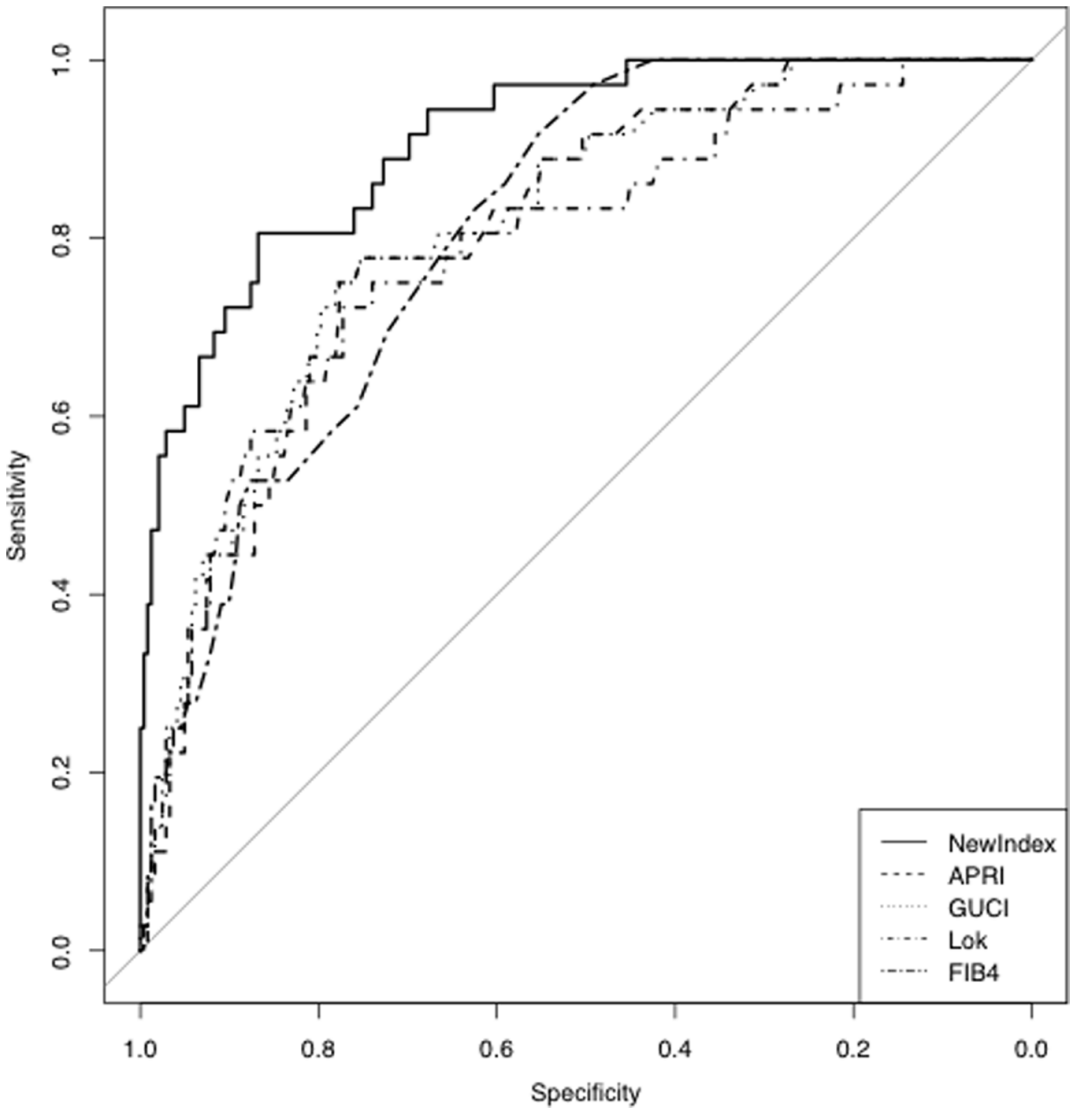
Receiver operator characteristics curves plotting sensitivity against specificity for prediction of cirrhosis in the exploratory set (n = 278) for the new index (NoLI) in comparison with some other biochemical indices. The AUROC for NoLI was 0.91 (95% CI 0.86–0.96). The corresponding AUROC for the other indices in the exploratory set were for FIB4 0.81 (95% CI 0.75–0.87), Lok 0.79 (95% CI 0.71–0.87), APRI 0.81 (95% CI 0.74–0.88), and for GUCI 0.81 (95% CI 0.74–0.88).

The ROC curve formed by plotting sensitivity against specificity for the new index in prediction of cirrhosis in the exploratory set is shown in Figure 2. The area under the ROC curve (AUROC) was 0.91 (95% CI 0.86–0.96). Figure 2 also displays ROC curves for APRI [11], Lok index [10], GUCI [15] and FIB-4 [16]. Theses indices were chosen for comparison since they are all freely available non-invasive scores, evaluated and validated for the detection of cirrhosis. The corresponding AUROC for the other indices in the exploratory set were for FIB-4 0.81 (95% CI 0.75–0.87), Lok index 0.79 (95% CI 0.71–0.87), APRI 0.81 (95% CI 0.74–0.88), and for GUCI 0.81 (95% CI 0.74–0.88).

### Cutoff Values

**Figure 3:**
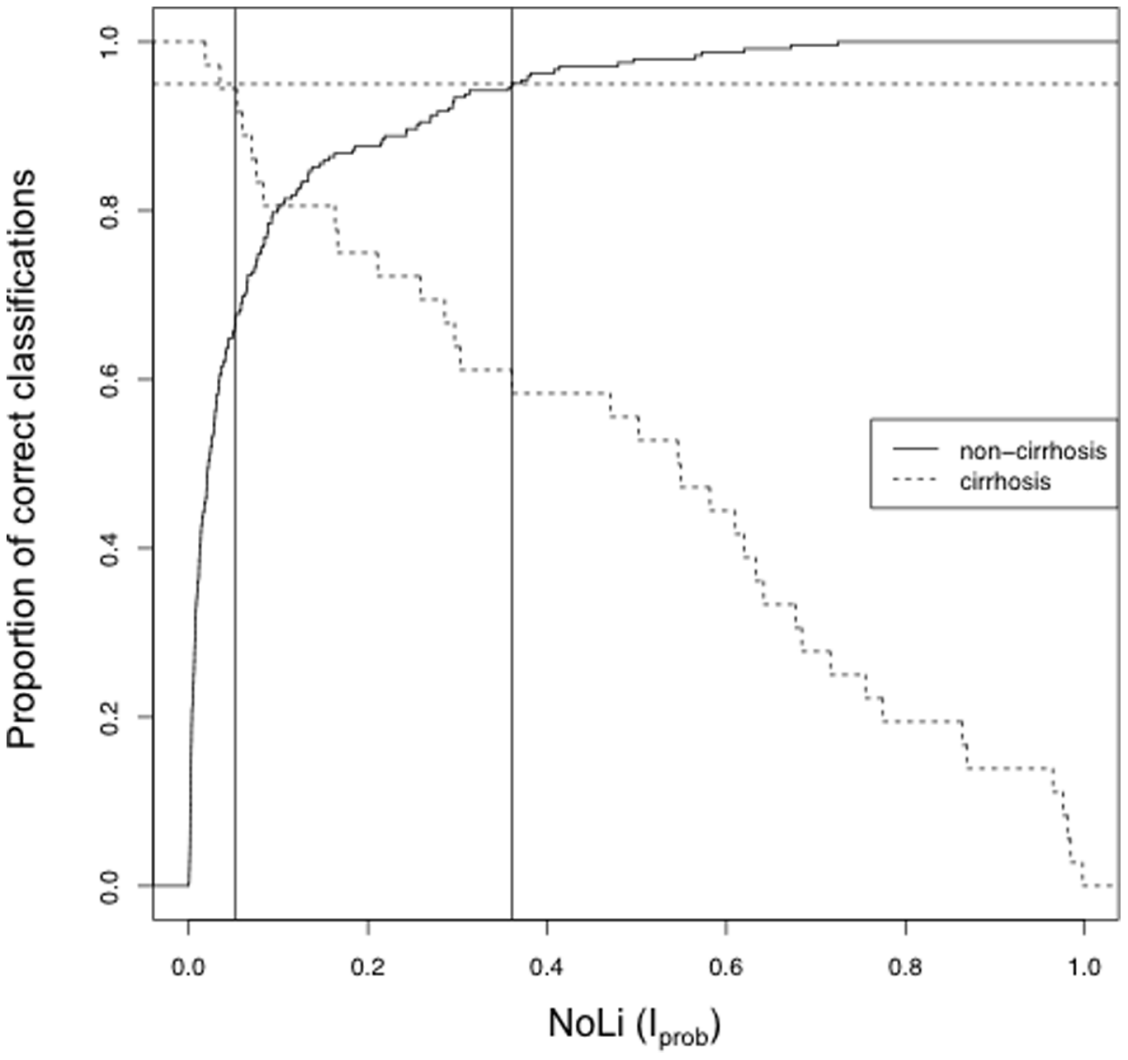
Proportion of correct classifications (i.e. non-cirrhotic classified as non-cirrhotic and cirrhotic classified as cirrhotic) according to different cut-off levels for NoLI. The solid vertical lines represent the chosen cut-offs 0.054 and 0.37. Using these cut-offs, the misclassification error would be approximately 5% for cirrhotic as well as non-cirrhotic patients, respectively (dotted horizontal line).

Two cut-off values were chosen that would produce a minimal misclassification error of approximately 5% for each group. Using a predicted cut-off value of <0.053 for the exclusion of cirrhosis and >0.37 for the identification of cirrhosis would misclassify 5.6% (2/36) of patients with cirrhosis as non-cirrhotic and 5.0% (12/242) of non-cirrhotic as having cirrhosis (with a cirrhosis prevalence of 13%, a misclassification rate of 5.6% is the closest to 5% we can get). Figure 3 shows the proportion of correct classifications for cirrhosis and non-cirrhosis according to different cut-offs. The sensitivity, specificity, likelihood ratios and negative and positive predictive values for the proposed cut-offs (0.053 and 0.37) are displayed in table 2. Among the 12 patients misclassified as having cirrhosis, the Ishak fibrosis stages were distributed as follows; F4 n = 7 (58%), F3 n = 3 (25%) and F2 n = 2 (17%). HCV genotypes 2 and 3 were evenly distributed.

**Table 2:** Diagnostic accuracy for the proposed cut-off levels regarding the NoLI score for prediction of cirrhosis in 278 HCV-infected patients.

Cut-off	Sensitivity (%)	Specificity (%)	+LR	−LR	NPV (%)	PPV (%)
0.054	94(81–99)	68(62–74)	2.9(2.4–3.6)	0.08(0.02–0.32)	99(96–100)	30(22–40)
0.37	58(41–75)	95(92–97)	10.9(6.0–19.7)	0.44(0.30–0.65)	94(90–97)	62(44–78)

Abbreviations: +LR, positive likelihood ration; −LR, negative likelihood ratio; NPV, negative predictive value; PPV, positive predictive value. All values are presented with 95% confidence interval.

### Validation Set

**Figure 4:**
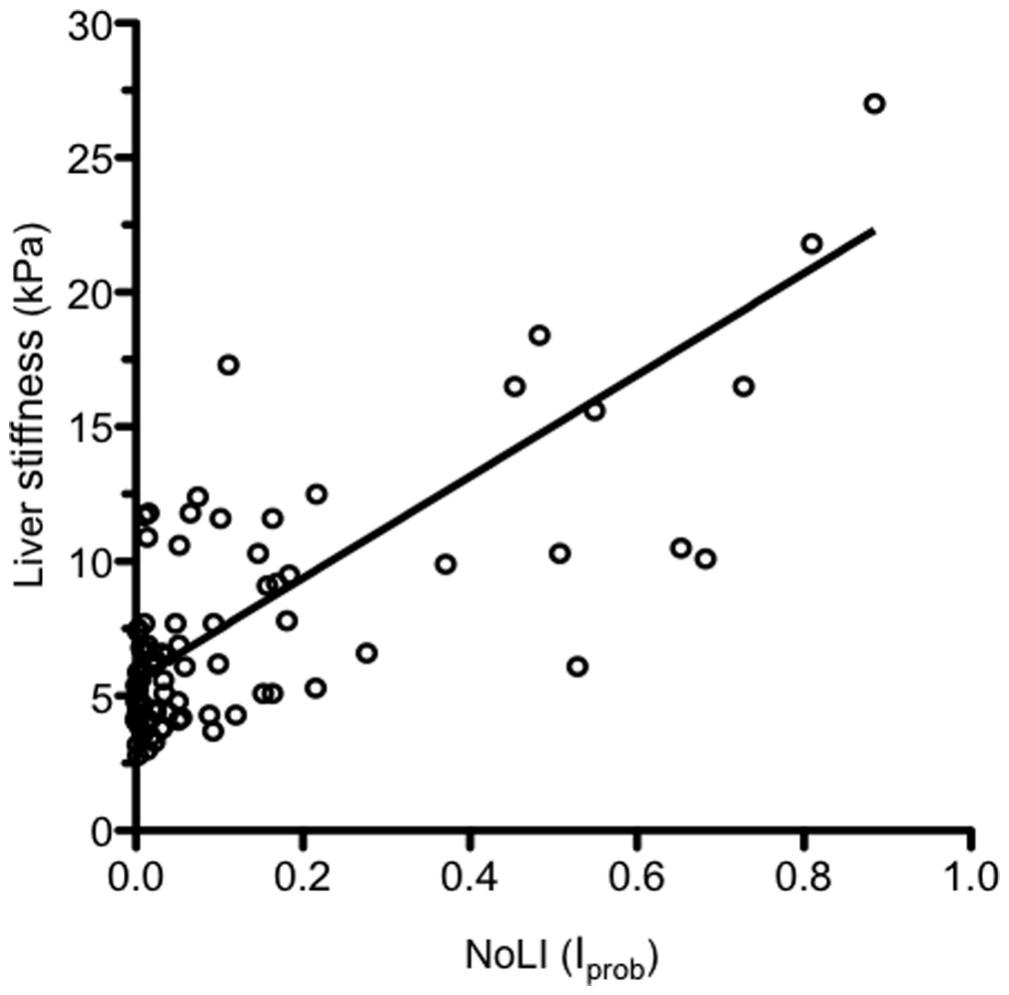
Scatter plot comparing the New index (NoLI) with liver stiffness measured by transient elastography. The Spearman correlation coefficient was 0.542 (p<0.001).

Applying the new index to the validation set, the AUROC for prediction of cirrhosis was 0.90 (95% CI 0.83–0.98). In the validation set, patients were also examined by transient elastography. The Spearman correlation coefficient for the new index and liver stiffness values was 0.54 (p<0.001; Figure 4). One patient (a 66 year-old genotype-1-infected male with compensated cirrhosis and mild steatosis) with a liver stiffness value of 50 kPa was included in the calculation but not in the figure. To evaluate the diagnostic capability of the new index using transient elastography as reference, we used a cut-off of 12.5 kPa for cirrhosis [17]. The resulting Roc-curve had an AUROC for prediction of cirrhosis of 0.95 (95% CI 0.89–1.0).

Since the exploratory set included only patients infected with HCV genotypes 2 and 3 and the validation set mainly genotype 1 infected patients, we combined the exploratory and validation set to evaluate potential genotypic differences. No significant differences were detected and the AUROC for specificity vs. sensitivity for each genotype was 0.91 (95% CI 0.82–1.0), 0.86 (95% CI 0.73–0.98) and 0.93 (95% CI 0.88–0.97) for genotypes 1, 2, and 3 respectively.

## Discussion

The primary aim of this study was to develop a reliable model for predicting HCV-related liver cirrhosis. The model created could confidently predict cirrhosis in both the exploratory and the validation sets, although the findings need to be confirmed in larger series. Although there is considerable overlap between adjacent fibrosis stages, this model has a good ability to exclude cirrhosis in patients with less severe stages of fibrosis (Ishak F0-4) as well as to predict cirrhosis in biopsy-verified cirrhotic patients (Ishak F5-6). Like many other biochemical indices, NoLI performs better in excluding than confirming cirrhosis.

The new index combines age, BMI, platelet count and prothrombin index, i.e. factors that previously have been consistently associated with fibrosis [6], [9]–[11], [35], along with D7-lathosterol which, to our knowledge, has not previously been evaluated in this setting. Serum D7-lathosterol is a precursor of cholesterol and its plasma concentration reflects cholesterol synthesis and correlates with 5-alfa-HMG-reductase activity, the rate-limiting enzyme of cholesterol synthesis [24], [25]. With the development of liver cirrhosis, the synthetic function in the liver, including cholesterol synthesis, decreases [36]. Thus, it is plausible that the concentration of D7-lathosterol in serum would reflect liver function and that decreased levels of lathosterol could be an early sign of advanced liver fibrosis. On the other hand, the sterols primarily reflecting cholestasis did not predict cirrhosis in this setting, which is not surprising since cholestasis is not a common feature in HCV-related cirrhosis. Accordingly, none of the cirrhotics in any of the sets cohorts had elevated serum-bilirubin levels indicating cholestasis. The hepatitis C virus is known to interfere with host lipid metabolism resulting in reduced levels of s-cholesterol and hepatic steatosis [37] that resolves following successful antiviral treatment, at least in genotype 3 infected patients [38], [39]. In non-genotype 3 patients, however, steatosis has been associated with insulin resistance and the metabolic syndrome [40], [41]. In a recent study, Clark et al [42] demonstrated that HCV genotype 3, but not genotype 2, interferes with the distal cholesterol pathway, which resolves after successful antiviral treatment. In the same study, no significant differences in the levels of lathosterol was found between genotype 2 and 3 infected patients suggesting that this genotype-specific interaction occurs further down in the pathway. The evaluation of NoLI index according to HCV genotype in the present study showed no statistically significant differences regarding AUROC between genotypes and the level of serum lathosterol does not seem to be genotype-dependent in this setting.

Several studies on the prediction of cirrhosis have been reported during the past 10 years [7], [10]–[13], [15], [16], [43]–[45]. In a large multicenter study by Degos et al [14] evaluating the diagnostic accuracy of Fibroscan and FibroMeter, FibroTest, APRI and Hepascore for the prediction of cirrhosis, AUROC ranged from 0.77 to 0.86. FibroMeter [7], FibroTest [14] and Hepascore [12] are all validated and frequently used for the prediction of cirrhosis but they are all protected by patented formula making them less accessible. The AST-to-platelet ratio index (APRI) [11] as well as the Lok-index [10] and FIB-4 [16] are all free of cost and based on routine laboratory parameters making them easy to use in routine clinical practice. When compared in the exploratory set, our model performed slightly superior to these other non-patented scores. Unfortunately, the size of and cirrhosis prevalence in the validation set did not allow for in-depth comparison.

Our study had some potential limitations. Measurement of serum lathosterol is not a standard biochemical test. Although it is measured using gas-chromatography standard patent-free laboratory technique, commonly used for analysis of other compounds, it may be slightly cumbersome to use. Still, the presence of D7-lathosterol improves the diagnostic performance of the NoLI index why we choose to keep it in the model.

The prevalence of cirrhosis in the exploratory and validation sets was 13% and 10% respectively, which is low compared with some other studies on non-invasive fibrosis markers using liver biopsy as reference [10], [21]. This lessens the ability to create a solid model for prediction. However, a cirrhosis prevalence of around 15% seems to be common in unselected Hepatitis C populations [14], [46]. The limited sample size and the low prevalence of cirrhosis in the validation set is a major limitation and does not permit accurate comparison between different indices, and the diagnostic performance of the new index need to be tested also in other larger cohorts. Moreover, other studies of fibrosis markers have been validated in cohorts of similar size, e.g. AST to platelet ratio index (APRI), a very reliable and widespread fibrosis index, was created in an exploratory set of 192 patients (15 (16%) cirrhotics) and validated in a cohort of 78 subjects (13 (17%) cirrhotics) [11]. The patients in the validation set were recruited from a group of consecutive patients referred for routine liver biopsy and were thus potential candidates for non-invasive fibrosis assessment in clinical practice. The patients in the exploratory and validation sets differed regarding HCV genotypes as well as country of residency, although none of these factors were independently associated with cirrhosis when patients in the two sets were analyzed together (cirrhosis was more common in Sweden and Denmark which was due to an age effect, data not shown). Thus they would not have influenced the statistical model.

In the exploratory set, liver biopsies and serum samples were not taken at the same time point, although both were sampled prior to initiation of therapy. The index, however, performed well in the validation set where blood samples were drawn on the day of the liver biopsy. As with all non-invasive tests, the use of liver biopsy as comparison poses a problem since liver biopsy is prone to sampling error and thus can both over- and underestimate the true liver fibrosis stage [4]. As shown by Bedossa et al only 65% of biopsies relying on 15-mm samples led to correct diagnosis using METAVIR scoring system [5]. Hence, the absolute correctness of a non-invasive fibrosis marker would need to be estimated by use of other methods. Potential exclusion of cirrhotic patients due to a greater tendency towards inadequate biopsy size may have introduced a bias in the exploratory set. However, contrary to this notion, non-invasive markers (GUCI and APRI) did not differ significantly among excluded (n = 27) vs. included patients (p = 0.3). Although not the primary aim of this study, it is still interesting to note that the concordance of this index with Transient Elastography might be greater than with liver biopsy (AUC 0.95 (95% CI 0.89–1,0) vs. 0.90 (95% CI 0.83–0.98). Another perhaps more appropriate endpoint to use as gold standard would be the risk of liver related complications or death over time but that would be beyond the scope of the present study. A liver biopsy can provide much more pertinent information than serologic fibrosis markers on liver histology and may remain of importance in some cases in the future. However, we believe that serologic markers should be considered an important complement to liver biopsy.

In summary, our results indicate that an index combining well-known predictors of liver fibrosis in combination with measurement of a non-cholesterol sterol can be useful in predicting cirrhosis. These new findings could be of value as a supplement to already existing non-invasive methods and the application of our model potentially could aid clinical decision-making, e.g. on which patients require continued monitoring in spite of successful antiviral treatment.

## Supporting Information

Table S1 **Summary of the final model for prediction of cirrhosis.**
(DOCX)
