# Supplementary material for: A Novel Fibrosis Index Comprising a Non-Cholesterol Sterol Accurately Predicts HCV-Related Liver Cirrhosis
Source: PLoS One. 2014 Apr 3;9(4):e93601. doi: 10.1371/journal.pone.0093601 (PMC3974766; doi:10.1371/journal.pone.0093601)
Supplement: Table S1 — Summary of the final model for prediction of cirrhosis. (DOCX) [file pone.0093601.s001.docx]

**Supplementary table S1. Summary of the final model**

| **Coefficients** | **Estimate** | **Standard Error** | **p-value** |
| --- | --- | --- | --- |
| (Intercept) | -12.174 | 3.63 | 0.0008 |
| Age | 0.110 | 0.03 | 0.0002 |
| BMI | 0.232 | 0.06 | 8.0 x 10^-5^ |
| D7-lathosterol | -0.013 | 0.006 | 0.02 |
| Platelets | -0.018 | 0.004 | 7.0 x 10^-5^ |
| Prothrombin-INR | 3.687 | 2.382 | 0.122 |

Log-odds (predicting cirrhosis) = -12.17 + (age x 0.11) + (BMI (kg/m^2^) x 0.23)

+ (D_7_-lathosterol (µg/ 100 mg cholesterol) x (-0.013)) + (Platelet count (x10^9^/L) x (-0.018))

+ (Prothrombin-INR x 3.69)

Predicted probability = exp (log-odds)/ (1+exp (log-odds))
